# Mid-term clinical and functional outcomes after reverse shoulder arthroplasty with latissimus dorsi transfer

**DOI:** 10.1186/s10195-026-00902-0

**Published:** 2026-01-30

**Authors:** Antonio G. Colombini, Peter Rab, Arno A. Macken, Madu N. Soares, Michael Kimmeyer, Igor J. Shirinskiy, Ion-Andrei Popescu, Laurent Lafosse, Geert Alexander Buijze, Thibault Lafosse

**Affiliations:** 1 Alps Surgery Institute, Clinique Générale Annecy, Annecy, France; 2 Department of Orthopaedics, Ospedale Montecchi di Suzzara, Mantua, Italia; 3 Department of Sports Orthopaedics, Technical University of Munich, Ismaninger Strasse 22, 81675 Munich, Germany; 4 Department of Orthopaedic Surgery and Sports Medicine, Erasmus Medical Centre, Rotterdam, The Netherlands; 5 Shoulder and Elbow Unit, Joint Research, Department of Orthopaedic Surgery, OLVG, Amsterdam, The Netherlands; 6 Department of Human Movement Sciences, Vrije Universiteit Amsterdam, Amsterdam, The Netherlands; 7 Department of Orthopaedic and Trauma Surgery, University Medical Centre Mannheim, Mannheim, Germany; 8 Romanian Shoulder Institute, ORTOPEDICUM – Orthopaedic Surgery and Sports Clinic, Bucharest, Romania

**Keywords:** Reverse shoulder arthroplasty, Latissimus dorsi transfer, Combined loss of elevation and external rotation, External rotation deficit, Activities of daily living, Mid-term results

## Abstract

**Background:**

Although reverse total shoulder arthroplasty (rTSA) with concomitant latissimus dorsi transfer (LDT) has been shown to effectively treat external rotation (ER) deficits, there are limited data regarding its outcomes with modern implants and its impact on activities of daily living (ADLs) requiring ER. The purpose of this study was to assess the mid-term clinical and radiographic outcomes of rTSA with concomitant isolated LDT in patients with an ER lag sign and posterior rotator cuff deficiency.

**Methods:**

This retrospective cohort study with prospective follow-up included consecutive patients who underwent rTSA with concomitant isolated LDT between 2010 and 2022 with a minimum follow-up of 2 years. Primary outcomes included the resolution of ER lag sign, active ER, and the Activities of Daily Living and External Rotation (ADLER) score. Secondary outcomes included the Auto-Constant Score (CS), Subjective Shoulder Value (SSV), activities of daily living requiring internal rotation (ADLIR) score, visual analog scale (VAS) for pain, and radiographic analysis of standardized radiographs.

**Results:**

In total, 32 procedures in 32 patients were identified. Of these, 22 procedures in 22 patients (68% female, 72.9 ± 8.4 years at surgery) were available for follow-up at 4.8 ± 2.2 years postoperatively (response rate 73%). The ER lag sign resolved in 95.5% of patients, the active ER improved significantly from −13° (−20–0°) preoperatively to 10° (0–20°) postoperatively (*p* = 0.002). The ADLER score increased from 20 (18–21.5) to 30 (28–30, *p* < 0.001). The CS improved from 32 (25–52) to 71 (67–75, *p* < 0.001) and the SSV from 30 (28–40) to 80 (65–100, *p* = 0.002), and low pain levels were reported. Internal rotation (*p* = 1) and the ADLIR score (*p* = 0.56) did not improve or decrease. No revisions or complications were observed.

**Conclusions:**

rTSA with concomitant isolated LDT resulted in favorable clinical, functional, and radiographic mid-term outcomes, with a high rate of resolved external rotation lag sign and a significant improvement in activities of daily life that require ER. This procedure should be considered a viable treatment option in patients undergoing rTSA with posterior rotator cuff deficiency and an ER lag sign.

*Level of evidence*: IV, retrospective case series.

## Introduction

In an increasingly aging population, reverse total shoulder arthroplasty (rTSA) is an established treatment option for restoring shoulder function in patients with cuff tear arthropathy (CTA) and massive irreparable rotator cuff tears (miRCT) [1–5]. Following rTSA, forward elevation and patient-reported outcomes have been reported to improve consistently, whereas restoration of external rotation (ER) is less reliable [3, 6–8]. Preoperative ER deficits due to posterior rotator cuff insufficiency, particularly with teres minor fatty infiltration, may not always be compensated by deltoid fiber recruitment and are associated with inferior clinical and functional outcomes following rTSA [9–11]. Moreover, while active ER can be measured clinically, its impact on limitations in activities of daily living (ADL) has yet to be fully established [12–14]. To assess the impact of an ER deficit on ADL, the activities of daily living requiring external rotation (ADLER) score has been proposed as an additional tool for the assessment of clinically relevant deficits in ER [12]. However, data on the ADLER score of patients undergoing rTSA with or without concomitant tendon transfer remain limited [13, 15, 16].

Latissimus dorsi tendon transfer (LDT), initially described for the treatment of obstetric brachial plexus injury [17], has been adapted to treat irreparable posterosuperior rotator cuff tears [18, 19]. Subsequent modifications have been made, either isolated or in combination with teres major (TM) transfer, for utilization in rTSA in the context of posterior rotator cuff deficits [9, 20, 21]. However, outcomes of tendon transfer augmentation to improve ER in the setting of rTSA have been variable and remain inconsistent [13, 21–23]. Although satisfactory clinical and radiographic outcomes have been reported for combined rTSA with concomitant LDT, there remains a paucity of comparative data in patients with combined loss of elevation and ER (CLEER) [13, 21–23]. Moreover, only a few studies to date have reported clinical outcomes of modern rTSA systems with concomitant LDT, with a further paucity of data on ADLs requiring ER, with existing studies also being limited by small sample sizes, ranging from only 9 to 21 patients [24–30]. The purpose of this study was to assess the mid-term functional, patient-reported, and radiographic outcomes of rTSA with LDT. On the basis of currently available evidence, it was hypothesized that this procedure would result in satisfactory clinical and radiographic outcomes and enable ADLs which require ER of the shoulder [24–29].

## Materials and methods

This was a retrospective cohort study with prospective follow-up, approved by the institutional ethics committee and performed according to the Declaration of Helsinki. All consecutive patients that underwent implantation of rTSA with concomitant isolated LDT, performed in a single center between January 2010 and January 2022 with a minimum follow-up of 2 years, were eligible for inclusion. Patients with less than 2 years of follow-up were excluded from this study. Patients were invited to attend a follow-up visit, which included a clinical assessment, standardized radiographic imaging, and patient-reported outcome measures (PROMs).

### Patient selection

Surgery was generally indicated in patients with an ER lag sign and CLEER together with the presence of fatty infiltration of the infraspinatus and teres minor Goutallier grade 3 or higher, as measured on computed tomography scans [31]. Patients with an intact posterior rotator cuff, with neurological impairment of the deltoid muscle, and without a preoperative ER lag sign were excluded from undergoing RSA with concomitant LDT.

### Surgical technique

During the follow-up period, the prosthesis models Delta Xtend™ (DePuy Synthes, Raynham, MA, USA), Tornier Aequalis Reversed™ (Tornier SASWright Medical Inc., Bloomington, MN, USA), and Tornier Aequalis Ascend™ (Tornier SASWright Medical Inc., Bloomington, MN, USA) were implanted. Surgery was performed under general anesthesia with patients positioned in the beach chair position as reported previously [16]. An uncemented humeral stem was implanted unless bone quality required cemented fixation. The proximal pectoralis major tendon was partially released to expose the LD. The LD was then dissected from its insertion and secured with two Orthocord (DePuy Mitek, Norwood, MA) non-absorbable sutures using a Krackow stitch. The TM insertion was preserved and protected with Cobb elevators. Subsequently, the armed LD tendon was then mobilized by blunt dissection under gentle traction. After creating a passage around the humerus using dissectors, the latissimus dorsi tendon was carefully passed and then tensioned. Fixation was performed at the lateral crest of the bicipital groove at the height of its original insertion with transosseous sutures or with Pec buttons (Arthrex, Inc., Naples, FL), depending on bone quality. Bony increased offset reverse shoulder arthroplasty (BIO-RSA) was performed in case of severe medialization of the glenoid articular surface.

After surgery, patients adhered to a standardized postoperative physical therapy protocol. This included the use of a 15° abduction brace for six weeks. Passive range of motion exercises were started after 3 weeks, and active range of motion exercises were commenced at 6 weeks postoperatively.

### Outcome variables

PROMs were recorded preoperatively as well as at minimum 2 years postoperatively. The primary outcomes included the ADLER score [20], active ER, the presence of an ER lag sign [32], and Hornblower’s sign [33]. Secondary outcome measurements comprised of the Auto-Constant Score (CS), Subjective Shoulder Value (SSV), the activities of daily living requiring internal rotation (ADLIR) score [34], and the visual analog scale (VAS) for pain [35, 36]. A satisfactory correlation between self-reported CS and clinician-assessed CS has been demonstrated previously [35]. Additionally, clinical assessment was performed to obtain passive and active range of motion (ROM). Chart review of electronic patient files was performed to obtain patient demographic data and surgery characteristics.

Radiographic analysis at the latest follow-up was performed using standardized anteroposterior as well as Y-view radiographs, when patients consented to undergo imaging. Radiolucent lines around the glenoid were classified according to Lazarus et al. [37] and around the humeral stem according to Schoch et al. [38]. Scapular notching was graded according to the Sirveaux Classification [11]. Moreover, osteolysis and fractures at the insertion site of the LD tendon were recorded. The radiographs were measured and graded by two independent authors in a standardized manner (A.G.C. and P.R.). Any conflicts were resolved by the senior author. Finally, revisions and complications were recorded, with patients who underwent a revision during the follow-up period excluded from the analysis.

### Statistical analysis

Statistical analysis was performed according to a predefined plan and using RStudio (RStudio Public Benefit Corporation, Boston, USA) and R version 4.2.3 (R Foundation for Statistical Computing, Vienna, Austria). Histograms and the Shapiro–Wilk test were used to assess normality of numerical data. In case of normal distribution, data are presented as mean and standard deviation, while median and interquartile range (IQR) are used to represent non-normally distributed data. Categorical data are presented numerically, with the corresponding proportions indicated. Comparisons of numerical data across different time points were performed using the paired *t*-test for normally distributed variables and the Wilcoxon signed-rank test for non-normally distributed variables. Subgroup analyses of clinical outcomes were performed using the unpaired *t*-test for normally distributed variables and the Mann–Whitney-*U* test for non-normally distributed variables. Categorical data were compared at different time points using McNemar’s test. For the radiographic evaluation, the interrater reliability among the two observers was assessed using Cohen’s kappa (*κ*) and interpreted according to Landis and Koch (≤ 0.20: slight agreement; 0.21–0.40: fair agreement; 0.41–0.60: moderate agreement; 0.61–0.80: substantial agreement; 0.81–1.0: almost perfect or perfect agreement) [39]. The significance level was set at 0.05, and all *p*-values were two-tailed.

## Results

**Fig. 1:**
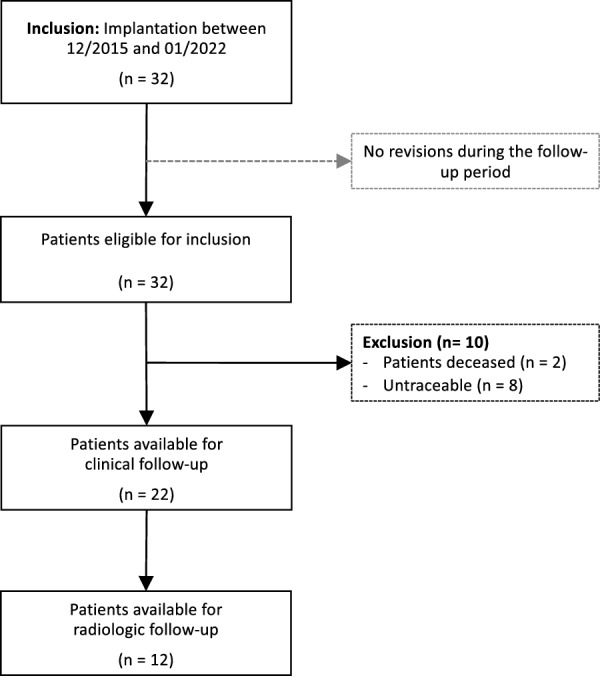
Flowchart of patient inclusion

In total, 32 consecutive procedures in 32 patients were identified between 2010 and 2022. Of these, 22 procedures in 22 patients were available for follow-up. The mean follow-up was 4.8 ± 2.2 years, with a response rate of 73%. The excluded cases consisted of two deceased patients (6.3%) and eight patients (25%) who could not be contacted (Fig. 1). No revision was performed during the follow-up period, and no complications were observed. The mean age at surgery was 72.9 ± 8.4 years, and 15 patients (68%) were female. CTA was the most common surgical indication, with the majority of patients receiving the Delta Xtend prosthesis model. The baseline characteristics are presented in Table 1.

**Table 1:** Study cohort characteristics

Study cohort characteristics (*n* = 22)	
Female, *n* (%)	15 (68.3)
Age at surgery, years ± SD	72.9 ± 8.4
Follow-up time, mean ± standard deviation	4.8 ± 2.2
Diagnosis, *n* (%)	
CTA	18 (81.8)
miRCT	3 (13.6)
Fracture sequelae	1 (4.5)
Implant type, *n* (%)	
Delta Xtend	20 (90.9)
Tornier Ascend Flex	1 (4.5)
Tornier Aequalis Reversed I	1 (4.5)
Glenoid component size, *n* (%)	
38	2 (9.1)
42	17 (77.3)
n.a	3 (15.0)
Humeral component size, *n* (%)	
10	6 (27.3)
12	11 (50.0)
14	2 (9.1)
n.a.	3 (15.0)
Cemented humeral component, *n* (%)	1 (4.5)
BIO-RSA, *n* (%)	3 (13.6)

*BIO-RSA* bony increased offset reverse shoulder arthroplasty, *CTA* rotator cuff tear arthropathy, *miRCT* massive irreparable rotator cuff tear, *n.a.* information not available, *SD* standard deviation

### Clinical and functional outcome

Postoperatively, the ER lag sign was resolved in 21 patients (95.5%). One patient (4.5%) exhibited a postoperative Hornblower’s sign. At final follow-up, the external rotation showed a significant increase of median 10° (IQR 0–22°) compared with the preoperative status (*p* = 0.002). Moreover, the ADLER score showed a statistically significant increase of median 8.5 (6.0–10) points (*p* < 0.001). Furthermore, a favorable shoulder function as well as low pain levels were reported, with a CS of 71 (67–75), which showed a significant improvement of median 39 (18–44) points compared with the preoperative score (*p* < 0.001). In addition, an anterior elevation of 165° (140–170°), an abduction of 160° (130–170°), and a median level reached with internal rotation of L3 (buttock–L1) were recorded (Table 2). Internal rotation and the ADLIR score did not significantly differ compared with the preoperative status (*p* = 1). No significant difference in ADLER score (30.0 [29.5—30.0] versus 30.0 [27.5–30.0], *p* = 0.53), active ER (5° [2.5–12.5°] versus 10° [5–25°], *p* = 0.49), or CS (71 [69–71] versus 71 [66–76], *p* = 0.63) was observed between patients with a lateralized glenoid (Tornier Ascend and/or BIO-RSA) and patients with a medialized glenoid.

**Table 2:** Preoperative and postoperative clinical and functional outcome measures

Clinical and functional outcome measurements			
	Preoperative	At final follow-up	*p*-Value
Primary outcomes			
Lag sign, *n* (%)	22 (100)	1 (4.5)	< 0.001
Hornblower ‘s sign, *n* (%)	4 (18.2)	1 (4.5)	< 0.001
Active external rotation, median (IQR)	−13 (−20–0)	10 (0–20)	0.002
ADLER, median (IQR)	20 (18–21.5)	30 (28–30)	< 0.001
Secondary outcomes			
Auto-Constant Score, median (IQR)	32 (25–52)	71 (67–75)	< 0.001
SSV, median (IQR)	30 (28–40)	80 (65–100)	0.002
VAS, median (IQR)	7 (6–7)	0 (0–1)	0.002
Active elevation, median (range)	90 (50–95)	165 (140–170)	< 0.001
Active abduction, median (range)	90 (55–100)	160 (130–170)	< 0.001
Active internal rotation, median (range)	L3 (buttock–L1)	L3 (buttock–L1)	1
ADLIR, median (IQR)	87 (83–93.5)	86 (81–90)	0.56

*ADLIR* activities of daily living which require internal rotation, *ADLER* activities of daily living which require external rotation, *IQR* interquartile range, *VAS* visual analog scale, *SSV* Subjective Shoulder Value

### Radiographic outcomes

**Fig. 2:**
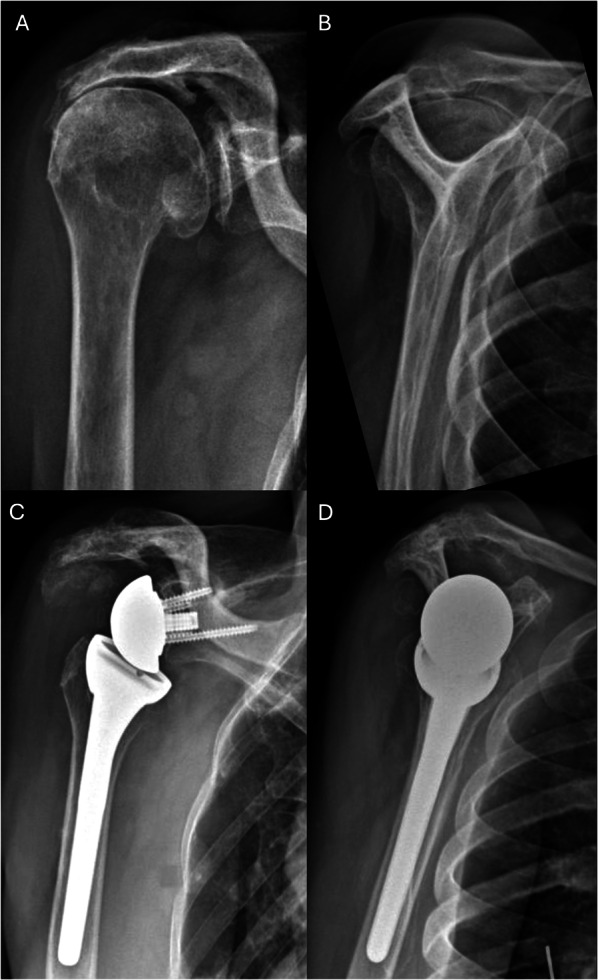
Clinical case of a 73-year-old female patient with cuff tear arthropathy and combined loss of elevation and external rotation (CLEER) undergoing reverse shoulder arthroplasty with concomitant latissimus dorsi transfer. **A**, **B** Preoperative anteroposterior and Y-view radiographs of a right shoulder. **C**, **D** Postoperative anteroposterior and Y-view radiographs

**Fig. 3:**
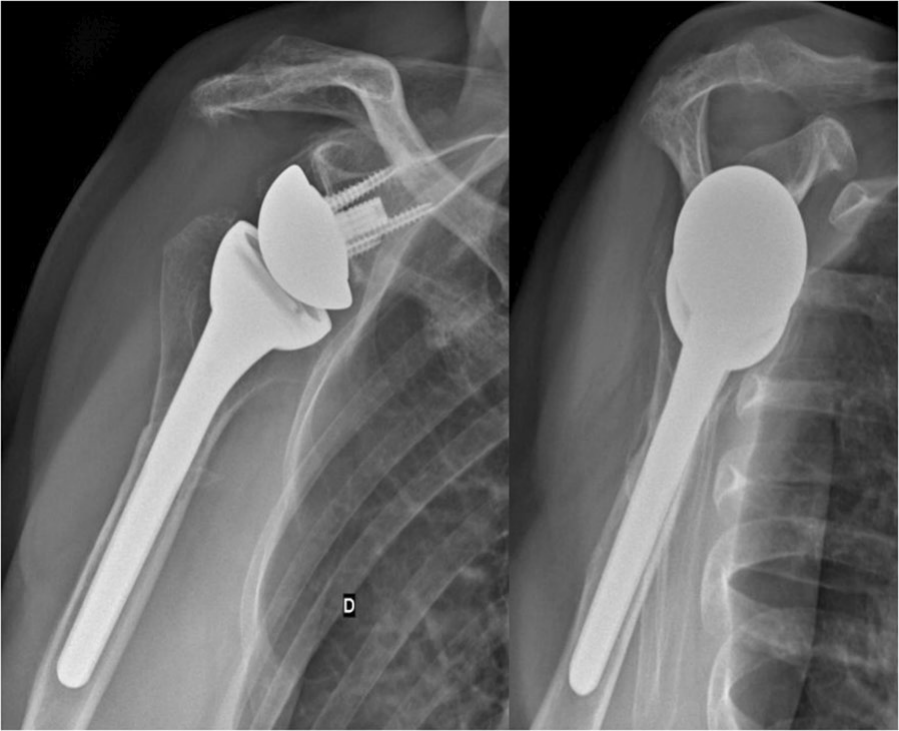
Radiograph of a right shoulder reverse shoulder arthroplasty with concomitant latissimus dorsi transfer with osteolysis at the latissimus dorsi tendon insertion site

Radiographic analysis was performed in 12 patients (54.5%; Fig. 2). Radiolucencies around the humeral stem were recorded in three patients (25%), and around the glenoid component in one patient (8.3%). No implant demonstrated high-grade radiolucencies or was considered at risk for definitive loosening. Osteolysis at the LDT site was observed in 42% of patients (5/12), occurring in 3 of 4 patients with button fixation and 2 of 6 patients with transosseous fixation (*p* = 0.52; Fig. 3). No patient showed signs of scapular notching, and one case (8.3%) of bone spurs of the acromion was recorded. No significant difference in ADLER score (29.0 [29.0–29.0] versus 26.0 [26.0–30.0], *p* = 0.73), active ER (5° [0–20°] versus 0° [0–13°], *p* = 0.61), or CS (71 [71–71] versus 71 [68–72], *p* = 1) was observed between patients with osteolysis at the LDT insertion site and patients without. The interrater reliability was moderate for radiolucencies around the glenoid (*κ* = 0.57) and humeral component (*κ* = 0.59), substantial for osteolysis at the LDT site (*κ* = 0.68), and almost perfect for notching (*κ* = 0.83) and bone spurs of the acromion (*κ* = 0.84). All disagreements in the radiographic analysis were discussed with the senior author, and the definitive agreement is reported.

## Discussion

This study aimed to report the clinical and radiologic outcomes of rTSA with concomitant LDT in patients with ER lag sign and combined loss of elevation and external rotation. The most important findings are the high rate of resolution of the ER lag sign (95.5%) and favorable clinical and functional outcomes observed at mid-term follow-up.

The favorable range of motion, along with the clinical and functional outcomes observed in this study, are consistent with existing literature that reports similarly positive results and significant improvements in ROM and patient-reported outcomes following rTSA with concomitant LDT with or without additional TM transfer [12, 16, 30, 40–44]. In an electromyographic study, Piedra et al. demonstrated that the transposed LD remains functionally active over a mean 6.7-year period [41]. To our knowledge, this mid-term case series represents one of the largest published to date, with previously reported sample sizes ranging from only 9 to 21 patients [30, 40]. Despite the favorable clinical outcomes reported, the translation of clinically measured ER into ER-related clinically relevant changes in daily life remains limited, in part owing to the underrepresentation of ER in traditional PROMs [8, 21, 45]. While the ADLER score aims to capture clinically relevant ER deficits [20], it has yet to be validated, and data on its use in patients undergoing rTSA with concomitant LDT remain limited. Nevertheless, observed ADLER scores align with previous findings [13, 15, 16, 20]. While no minimal clinically important difference (MCID) has been reported for the ADLER score, the increase in CS exceeds the MCID generally reported for rTSA [46–48]. However, despite the favorable clinical results reported in this study and in several systematic reviews [30, 40, 42], comparative studies evaluating patients with CLEER undergoing rTSA with or without additional LDT remain scarce. In a randomized controlled trial, Young et al. observed no significant differences in patient-reported outcomes, including the ADLER score, or range of motion between patients who received additional LDT and those who underwent rTSA alone [13]. Moreover, Wiater et al. observed similar results in a retrospective comparative case series [49]. However, substantial heterogeneity in patient age, follow-up duration, indications, and especially implant designs across studies may contribute to conflicting findings [21, 22, 30, 40].

In this study, the LD tendon was fixated at the lateral crest of the bicipital groove at the height of its original insertion. In the literature, substantial heterogeneity remains regarding fixation techniques and insertion sites of the LD tendon, and to date, no consensus has been established on the optimal insertion site [50–52]. Although high rates of osteolysis at the humeral stem insertion site have been reported previously [9, 53, 54], which is consistent with the findings of this study, the precise impact on implant stability and the risk of periprosthetic fracture has yet to be clearly established. In addition, there is considerable heterogeneity in surgical techniques, particularly with regard to the additional TM transfer, and no clear consensus has been reached as to whether its transfer confers a functional benefit [30, 50, 55]. In a comparative study, Kazum et al. reported no significant difference between patients undergoing rTSA with isolated LDT and additional transfer of the TM in patient-reported outcomes or ROM in 36 patients [55]. While the supplementary force vector of the transferred TM may offer potential benefits in restoring ER force and ROM, some authors suggest that it may result in a disbalance in force couples regarding ER and internal rotation, particularly in the absence of the subscapularis [16, 50, 51, 55, 56]. In this context, Flury et al. reported an internal rotation deficit for patients receiving LDT with TM transfer using a modified L’Episcopo technique [57]. In contrast, ADLIR scores and internal rotation showed no significant pre- to postoperative differences in this study, possibly owing to preservation of the TM. However, these hypotheses require further biomechanical and clinical validation.

Owing to substantial advancements in implant designs in recent years, some authors have proposed that a lateralized rTSA may be sufficient to restore ER in patients with ER deficits [58, 59]. In this context, while postoperative losses in ER have been reported in patients with medialized rTSA designs [60, 61], some authors have observed improvements in ER with the use of more lateralized implants [62]. However, data on patients with CLEER undergoing rTSA without concomitant LDT are limited. Berglund et al. reported favorable patient-reported outcomes in 24 patients with preoperative CLEER undergoing implantation or rTSA with a lateralized glenosphere design and a 135° neck shaft angle, but the postoperative presence of an ER lag or ADLs in ER was not recorded [63]. Moreover, a recent meta-analysis observed no superiority of rTSA with LDT compared with RSA with a lateralized glenoid or humeral component in CS and postoperative ER [59]. However, interpretation of the results may be influenced by factors such as inclusion of patients regardless of CLEER status, no distinction between humeral and glenoid lateralization, the absence of specific analyses for ADLER, ER lag, or Hornblower sign, and differences in preoperative ER [59]. As a result, there remains a paucity of data regarding whether a more lateralized prosthesis or further advancements in implant designs can effectively resolve ER deficits in patients with CLEER, particularly in terms of functional ER during ADLs. In this study, only one patient (with transosseous LDT fixation) did not experience postoperative resolution of the ER lag. This may be attributed to the aforementioned variations in fixation techniques and positioning, potential fixation failure, the absence of the subscapularis as a counteracting force [16, 55], or implant design, all of which are factors that warrant prospective evaluation.

## Limitations

The present study should be interpreted with the following limitations in mind: First, the small sample size and the lack of a control group may limit the statistical power of the study and the extrapolation of the findings to the general population. Owing to the retrospective nature of this study, a reliable comparison with patients undergoing rTSA without LDT could not be made. Further comparative studies are necessary to allow for definitive conclusions and implications for clinical practice. Second, bias related to the loss to follow-up cannot be ruled out. Third, the patients included in this study were heterogeneous in terms of surgical indications, implant type, LDT fixation techniques, and age. Fourth, radiographs with a minimum follow-up of 2 years were unavailable in some cases, which limited the reliability of the radiographic analysis. Fifth, the subgroup analyses of clinical and radiographic outcomes were limited by sample size constraints, and further comparative studies are needed to assess the influence of the implant type and osteolysis at the LDT site on outcomes following RSA with concomitant LDT. Finally, given the single-center nature of the study, the external applicability of its findings may be limited.

## Conclusions

RTSA with concomitant isolated LDT resulted in favorable clinical, functional, and radiographic mid-term outcomes, with a high rate of resolved ER lag sign and a significant improvement in activities of daily life that require ER. This procedure should be considered a viable treatment option in patients with posterior rotator cuff deficiency and ER lag sign. However, future comparative studies are required to determine the added value of an LDT with modern and lateralized prosthesis designs.

## Data Availability

All data generated or analyzed during this study are included in this published article.

## Abbreviations

ADL: Activities of daily living
ADLER: Activities of daily living requiring external rotation score
ADLIR: Activities of daily living requiring internal rotation score
BIO-RSA: Bony increased offset reverse shoulder arthroplasty
CLEER: Combined loss of elevation and external rotation
CS: Auto-Constant Score
CTA: Cuff tear arthropathy
ER: External rotation
IQR: Interquartile range
LDT: Latissimus dorsi tendon transfer
MCID: Minimal clinically important difference
miRCT: Massive irreparable rotator cuff tear
PROM: Patient-reported outcome measures
RC: Rotator cuff
ROM: Range of motion
rTSA: Reverse total shoulder arthroplasty
SSV: Subjective shoulder value
TM: Teres major
VAS: Visual analog scale
